# Plutonium in Manhattan Project workers: Using autopsy data to evaluate organ content and dose estimates based on urine bioassay with implications for radiation epidemiology

**DOI:** 10.1371/journal.pone.0259057

**Published:** 2021-10-26

**Authors:** Martin Šefl, Joey Y. Zhou, Maia Avtandilashvili, Stacey L. McComish, Sergei Y. Tolmachev

**Affiliations:** 1 United States Transuranium and Uranium Registries, College of Pharmacy and Pharmaceutical Sciences, Washington State University, Richland, Washington, United States of America; 2 Office of Domestic and International Health Studies, United States Department of Energy, Washington, DC, United States of America; Northwestern University Feinberg School of Medicine, UNITED STATES

## Abstract

**Purpose:**

Radiation dose estimates in epidemiology typically rely on intake predictions based on urine bioassay measurements. The purpose of this article is to compare the conventional dosimetric estimates for radiation epidemiology with the estimates based on additional post-mortem tissue radiochemical analysis results.

**Methods:**

The comparison was performed on a unique group of 11 former Manhattan Project nuclear workers, who worked with plutonium in the 1940s, and voluntarily donated their bodies to the United States Transuranium and Uranium Registries.

**Results:**

Post-mortem organ activities were predicted using different sets of urine data and compared to measured activities. Use of urinalysis data collected during the exposure periods overestimated the systemic (liver+skeleton) deposition of ^239^Pu by 155±134%, while the average bias from using post-exposure urinalyses was –4±50%. Committed effective doses estimated using early urine data differed from the best estimate by, on average, 196±193%; inclusion of follow-up urine measurements in analyses decreased the mean bias to 0.6±36.3%. Cumulative absorbed doses for the liver, red marrow, bone surface, and brain were calculated for the actual commitment period.

**Conclusion:**

On average, post-exposure urine bioassay results were in good agreement with post-mortem tissue analyses and were more reliable than results of urine bioassays collected during the exposure.

## Introduction

Radiation epidemiological studies, such as the Million Persons Study [1,2] and International Nuclear Workers Study [3], typically rely on worksite records and bioassay measurements to estimate intakes, systemic deposition of radionuclides and, ultimately, doses to the organs. For plutonium, the well-known cohorts for studying the effects of occupational exposure are Mayak workers (Russian Federation) [4–7], Sellafield workers (United Kingdom) [8,9], and workers from the United States Department of Energy sites [10–13]. Bioassay measurements are often available only for a fraction of exposed workers, or the number of bioassay measurements is limited, and the samples are typically collected during the employment period as part of radiation protection monitoring programs [4,14]. The radiation protection approach is often conservative and tends to overestimate intakes and doses to protect the workers’ health [15]. The calculation of the organ doses from bioassay relies on the biokinetic and dosimetric models of the incorporated radionuclide. The accuracy of biokinetic models can and should be evaluated, for example, by using measured activities in organs and tissues of workers collected at autopsy.

In August 1968, the United States Transuranium and Uranium Registries (USTUR) was founded as the National Plutonium Registry by the Atomic Energy Commission with the goal to correlate accidental intakes of plutonium with workers’ subsequent health records [16,17]. The USTUR is a unique resource of tissue samples collected from workers with known or suspected intakes of actinides that are radiochemically analyzed. Today, the USTUR stores samples and analysis results from 362 volunteer tissue donors. The tissue analysis results combined with the workers’ bioassay data and exposure histories from their worksite records are evaluated at the USTUR to better understand actinide retention and distribution in the human body.

In this study, post-mortem organ activities are used to examine how well the International Commission on Radiological Protection (ICRP) biokinetic models describe the available data. This was quantified as a bias, which is a relative difference between predicted and measured post-mortem organ activities. Urine bioassay measurements and post-mortem liver and skeleton activities were used simultaneously to calculate the best estimates of committed effective doses.

The methodology was applied on a pilot group of 11 former Manhattan Project workers, who had worked at Los Alamos Scientific Laboratory in the 1940s [18]. These 11 workers were among 26 Manhattan Project workers who were selected for medical follow-up by the worksite after the end of their employment since their urinalyses indicated high systemic plutonium deposition. They were informally called the UPPU (You Pee Pu) Club [19]. Summaries of their conditions after the follow-up exams were previously published [20–25].

## Materials and methods

### Case selection

This study was performed as a part of the USTUR research program, which was reviewed and approved by the Central Department of Energy (DOE) Institutional Review Board No. WASU-68-50181.

**Fig 1:**
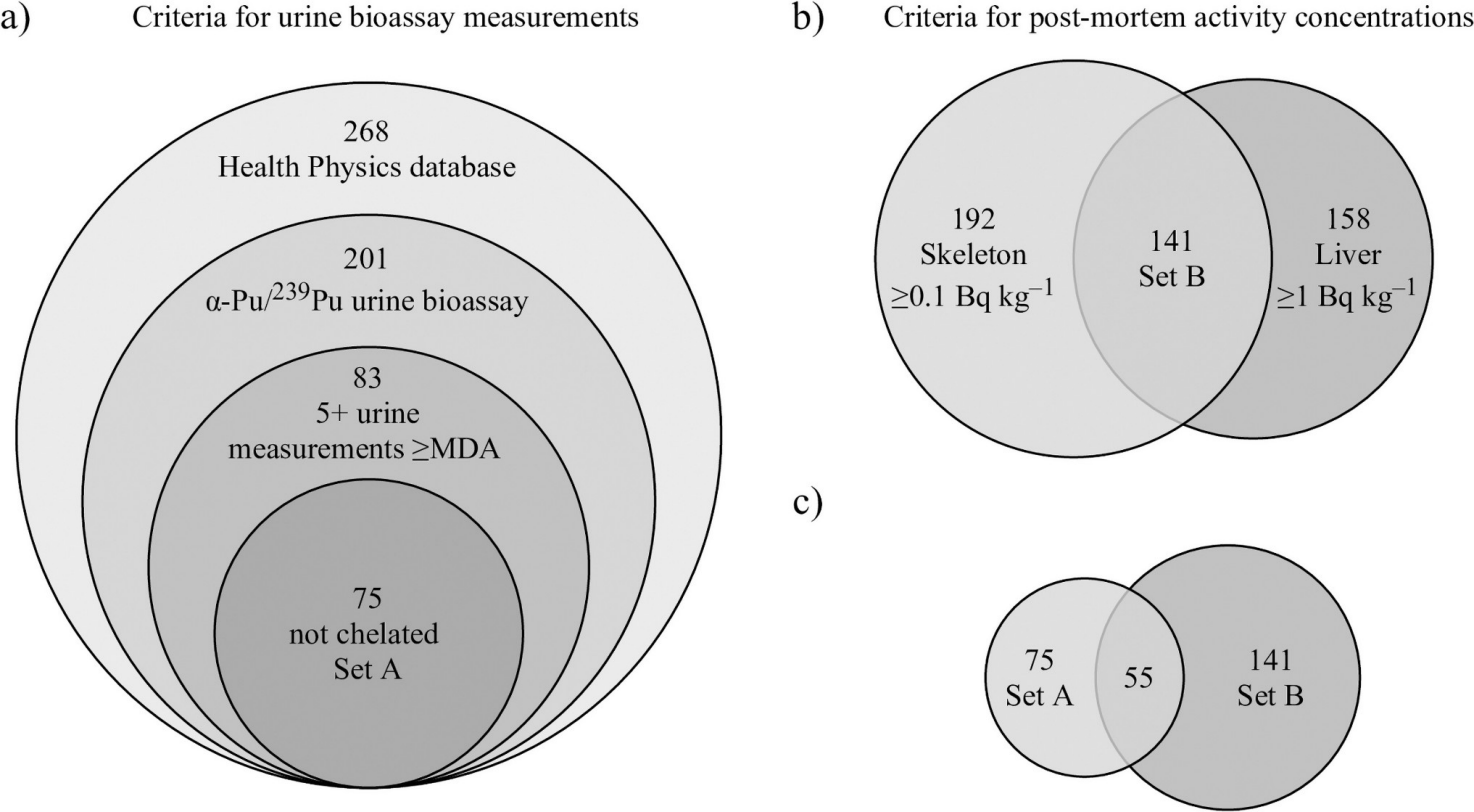
Diagram of case selection based on a) urine bioassay measurements, b) post-mortem plutonium activity concentrations in the liver and skeleton, c) both criteria.

The USTUR’s Health Physics and Radiochemistry databases were searched to select individuals for this study. Among 251 deceased USTUR Registrants, whose bioassay data were standardized in the Health Physics Database as of May 1, 2021, 201 cases had at least one record of a Pu or ^239^Pu urine bioassay measurement. Of those, only 83 cases had at least five urine measurements with the measured activity higher than contemporary Minimum Detectable Activity (MDA) provided in the technical basis documents for the National Institute for Occupational Safety and Health (NIOSH) Radiation Dose Reconstruction Program [26–29]. Eight of these 83 individuals underwent extensive decorporation therapies that significantly altered the biokinetics of incorporated Pu and, therefore, were excluded. This resulted in a list of 75 cases suitable for this study (Set A) (Fig 1a).

Furthermore, the USTUR Radiochemistry database search identified 192 Registrants who had ^239^Pu activity concentrations of more than 0.1 Bq kg^–1^ in their skeletons at the time of death, and 158 who had more than 1 Bq kg^–1^ in their livers. Intersection of these two sets resulted in 141 Registrants with high enough organ activity concentrations for this study (Set B, Fig 1b). The intersection of sets "A" and "B" resulted in a set of 55 cases (Fig 1c). Hence, these 55 individuals met both the criteria that a case had at least five urine samples with measured activity higher than the contemporary MDA and no extensive chelation (Set A) [27], and the criteria that a case met the above-mentioned thresholds for organ activity concentrations (Set B).

Among those 55 individuals, a special group of 11 individuals was further selected for this initial study. They were members of the ‘UPPU’ club, a group of 26 former Manhattan Project plutonium workers who were selected by the worksite health physics personnel for medical follow-up due to their high intakes of plutonium. The studied individuals worked with plutonium on average for 1.5±1.1 y and their average age at exposure was 25±6 y, ranging from 19 to 36 y. Two other USTUR cases from this group, Case 0669, and Case 0789, were not used in this study. For Case 0669, only a clavicle and seven soft tissue samples excluding liver were collected at autopsy, and for Case 0789, only lungs and thoracic lymph nodes were collected.

### Bioassay data

**Fig 2:**
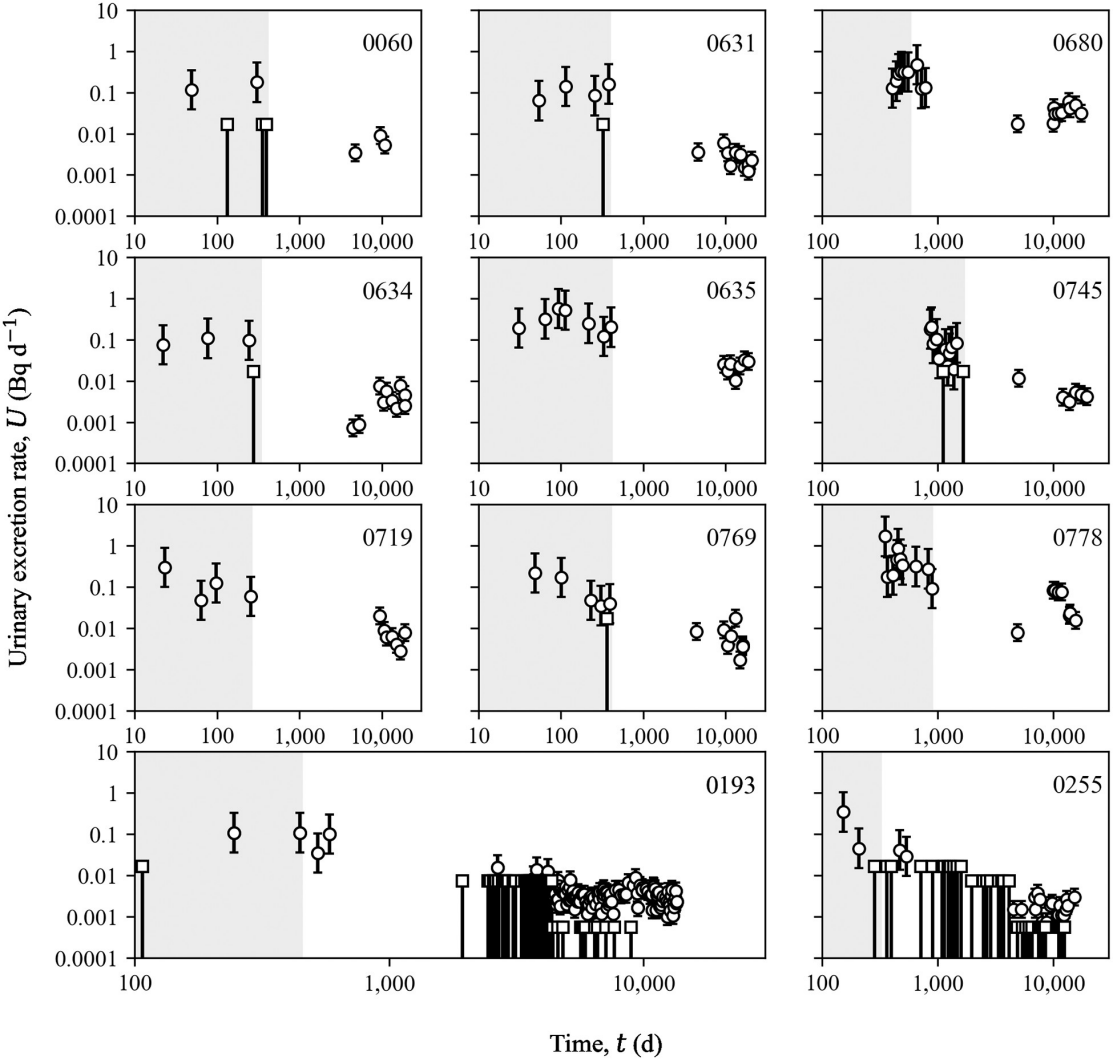
Urine measurements as a function of time after employment start date. Each chronic exposure period is indicated by the gray area (start day 0 not shown). Real measurements are displayed as circles with error bars above and below, <MDA/2 are displayed as squares with error bar below.

Urinary excretion rates for the selected 11 cases are provided as S1 Appendix and shown in Fig 2. Table 1 summarizes the total number of urine measurements for each case, how many of them were above MDA/2, and what was the percentage of >MDA/2 measurements. At Los Alamos, the MDA and precision/accuracy of urine sample analyses varied over time. In Registrant’s records, historical urine data typically lack specific information on the lower limit of detection, MDA, etc. Therefore, the median MDA values provided by NIOSH were used for this study [27]. Before October 1, 1949, urine was analyzed by a cupferron method, the MDA was high, and the precision and accuracy was low. Urine data after 1957 are believed to be a less important source of error due to improved measurement techniques [11].

**Table 1:** Summary of urine bioassay measurements.

Case	Number of measurements		Number of measurements by period		
	Total	>MDA/2	1945–1949	1949–1957	1958–1997
0060	8	5 (63%)	5 (62%)	0 (0%)	3 (38%)
0193	177	123 (69%)	5 (3%)	46 (26%)	126 (71%)
0255	57	23 (40%)	16 (28%)	8 (14%)	33 (58%)
0631	16	15 (94%)	5 (31%)	0 (0%)	11 (69%)
0634	14	13 (93%)	4 (29%)	0 (0%)	10 (71%)
0635	14	14 (100%)	7 (50%)	0 (0%)	7 (50%)
0680	19	19 (100%)	9 (47%)	0 (0%)	10 (53%)
0719	11	11 (100%)	4 (36%)	0 (0%)	7 (64%)
0745	19	17 (89%)	13 (68%)	0 (0%)	6 (32%)
0769	14	13 (91%)	6 (43%)	0 (0%)	8 (57%)
0778	18	18 (100%)	10 (56%)	0 (0%)	8 (44%)

### Post-mortem tissue activities

At the USTUR, tissue samples collected at an autopsy are dried at 120ºC, wet-ashed with a mixture of nitric acid and hydrogen peroxide, and dry-ashed at 450ºC. Then, samples are dissolved in hydrochloric acid. Chromatography and ion exchange techniques are used to separate ^239+240^Pu and ^238^Pu from other actinides. The isolated plutonium is electroplated, and ^239+240^Pu is measured by alpha spectrometry. The process was described elsewhere [30–33].

For all 11 cases, the liver activities were calculated from measured concentrations in analyzed samples and the autopsy weight of the whole liver. The determination of skeleton activities depended on available bone samples. The total skeleton activities for four whole-body cases (0193, 0635, 0680, 0769) were reported elsewhere [34]. For the remaining cases, the skeleton activities were estimated as a product of the skeleton weight and average activity concentration of measured bone samples (S2 Appendix). For whole-body cases, the skeleton weights were measured, for partial body cases, the skeleton weights were estimated using the following equation [35]

$$W_{\text{skel}}=-0.25+0.046H+0.036W-0.012A,$$ (1)

where *H* is body height in cm, *W* is body weight in kg, and *A* is an age in years. A summary of donation types and radiochemistry results is given in Table 2.

**Table 2:** Summary of the tissue radiochemical analysis results.

Case	Age at death (y)	Donation type	Skeleton				Activity (Bq)		Ratio
			Analyzed samples	*W*_skel_ (kg)	*C*_*skel*_ (Bq/kg)	Activity (Bq)	Liver	Skeleton +Liver	Skeleton-to-Liver
0060	50	PB^a^	5	10.26	6.96±1.30	71.4±13.4	54.6±1.8	126±14	1.31±0.25
0193	62	WB^b^	89	8.43	6.03±0.04	50.8±0.3	48.6+2.8	99±3	1.05±0.06
0255	70	PB	7	9.3	2.99±1.42	27.8±13.2	53.1±1.7	81±13	0.52±0.25
0631	85	WB	7^c^	7.96	14.5±4.6	116±36	91.8±1.9	207±36	1.26±0.40
0634	90+	WB	9^c^	9.14	20.6±5.8	188±53	229.9±5.3	418±53	0.82±0.23
0635	85	WB	82	11.82	65.2±0.2	770.8±2.8	927±17	1698±17	0.83±0.02
0680	90	WB	82	9.12	76.8±0.3	700.1±2.5	661±11	1361±11	1.06±0.02
0719	86	PB	5	9.16	18.9±3.9	173±36	259±5	432±36	0.67±0.14
0745	82	WB	28^c^	10.41	23.0±5.2	240±54	210±11	450±55	1.14±0.26
0769	66	WB	79	11.29	14.6±0.1	165.2±1.3	75.8±2.6	241±3	2.18±0.08
0778	66	PB	4	10.79	83.2±37.2	897±402	354±11	1250±402	2.53±1.14

^a^Partial body donation.

^b^Whole body donation.

^c^Only selected bones out of the entire skeleton were analyzed.

### Data analysis and biokinetic modelling

IMBA (Integrated Modules for Bioassay Analysis) Professional Plus^®^ version 5.0 [36,37] with the ICRP 130 Human Respiratory Tract model, ICRP 141 Plutonium Systemic model, and ICRP 30 Gastrointestinal Tract model was used to predict intakes and plutonium activity in the liver and skeleton at the time of death [38–40]. Biokinetic modelling of Case 0680, whose data was included in this study, was described in detail in a recent article [41]. All individuals investigated in this group had worked at the same worksite, and therefore, it was assumed they had been exposed to the same plutonium mixture with particle size of 0.3 μm AMAD (activity median aerodynamic diameter). A super-complex intake scenario (SCIR) with fixed material composition of 78% of Pu-nitrate and 22% of PuO_2_, which was derived for Case 0680 [41], was applied. SCIR is a mode in IMBA that allows the user to fit several intake regimes as one intake.

Table 2 indicates that the ratio of the activity in the liver and skeleton varied from 0.52±0.25 (Case 0255) to 2.53±1.14 (Case 0778). The sum of the liver and skeleton activities was used for intake predictions to serve as a robust measure of systemic plutonium activity [42]. For the purpose of calculations, the error in the liver+skeleton activity was assumed to follow a normal distribution with a relative standard deviation of 0.1 [41,43,44]. Urine bioassay errors were assumed to be distributed lognormally with a geometric standard deviation (GSD) of 3.0 for measurements before 1949, a GSD of 2.0 for measurements between 1949 and 1957, and a GSD of 1.6 for measurements after 1957 [45]. The less than MDA/2 urine bioassay measurements were replaced by MDA/2 and marked as ‘<LOD’ in IMBA. IMBA treats the value marked as ‘<LOD’ as an upper boundary to calculate probability that the true value is between zero and MDA/2. It was shown that this approach provides an unbiased intake estimate, if assumed uncertainty of the measurements is correct; however, if it is not, the bias increases with the percentage of ‘<LOD’ measurements [46]. Since sample specific detection limits were not available, using MDA/2 leads to overestimation of the intake. An MDA/2 of 0.0167 Bq/d was used for measurements before 1949; 7.4×10^−3^ Bq/d for measurements between 1949 and 1957; and 5.55×10^−4^ Bq/d for measurements after 1957 [27].

In a conventional dosimetric approach for radiation epidemiology, it is common to assume chronic intakes over the entire period of employment [47–49]. In the Mayak Worker Dosimetry System-2013, the chronic intake period covered the time when an individual worked in a plant with high risk of exposure [5,6], and the chronic exposures included a 3-step function of time with values high, medium, and low [50]. Furthermore, Puncher et al. showed that if intake time is unknown and all times are equally likely, a constant chronic intake provides an unbiased estimate of intake [51]. Therefore, in this study, chronic intakes over the entire period of employment were assumed. The exceptions to this rule were Case 0193 and Case 0255, who were employed at Los Alamos for 41 y and 37 y, respectively. These individuals had a highest potential of exposure to soluble plutonium only during the first two years of their employment while they were involved in the Manhattan Project. Their chronic intake periods were set according to their employment records, which indicated when they worked with plutonium material.

In the first part of this study, the post-mortem liver and skeleton activities predicted using urine measurements were compared to the activities measured after autopsy. The post-mortem organ activities estimated using urine bioassays were employed to examine how well the ICRP biokinetic models describe the data for this study group. To simulate a situation when only early monitoring bioassay data were available, urine bioassay measurements were divided into two subsets, 1) measurements collected during the chronic exposure period, denoted as U(E), and 2) measurements collected post-exposure denoted as U(P) (S1 Appendix). As a result, the post-mortem tissue activities were estimated three times using:

1. all *urine* data *A*_U_,
2. urine measurements that were collected during the *exposure* period only *A*_U(E)_,
3. urine measurements that were collected *post-exposure A*_U(P)_.

The bias of the predicted post-mortem activities in the liver and skeleton was calculated using the following equation

$$Bias\left(\%\right)=\frac{A_{j}-A}{A}\times 100\%,$$ (2)

where *A*_j_ was the predicted activity (*A*_U_, *A*_U(E)_, or *A*_U(P)_), and *A* was the measured post-mortem organ activity in the liver, skeleton, or liver+skeleton. The mean absolute bias was also calculated to characterize individual biases.

Committed effective doses, *E*(50), chosen as a measure of the effect of the plutonium intake on an individual, were calculated from the predicted intakes using ICRP 141 dose coefficients [38]. For the 78% Pu-nitrate and 22% PuO_2_ mixture with particle size of 0.3 μm AMAD, the dose coefficient was 38 μSv Bq^-1^.

In this study, committed effective doses were estimated for all 11 individuals using five subsets of available data:

1. the complete set of *urine* bioassay measurements available *E*_U_,
2. the subset of urine bioassay measurements that were collected during the *exposure* period, *E*_U(E)_,
3. the subset of urine bioassay measurements that were collected *post-exposure*, *E*_U(P)_,
4. the measured post-mortem *liver+skeleton* activity only, *E*_LS_,
5. both the *urine* bioassay and the post-mortem *liver+skeleton* activity, *E*_U+LS_.

Biases for committed effective doses were calculated for *E*_U_ and *E*_U(E)_ with *E*_U+LS_ as a reference value. Since all available data are accounted for in *E*_U+LS_, this was assumed to be the best estimate. The dose bias was calculated using the following equation

$$Bias\left(\%\right)=\frac{E_{j}-E_{U+LS}}{E_{U+LS}}\times 100\%,$$ (3)

where *E*_j_ represented *E*_U_ or *E*_U(E)_.

Radiation doses to selected tissues for the period from the beginning of intake to death were calculated using USTUR’s in-house code, which implements ICRP’s biokinetic models. Absorbed doses to the target tissues were calculated using the number of transitions in source regions (biokinetic compartments), energy released per one alpha transition of ^239^Pu, and specific absorbed fractions published by ICRP [52,53]. Equivalent and effective doses were calculated using ICRP recommended radiation and tissue weighting factors [54,55]. Our code was validated for ^239^Pu by calculation of 50-y committed equivalent doses using Taurus internal dosimetry software version 1.0 (Public Health England, Radiation Hazards and Emergencies Department, Chilton, Didcot, Oxon, UK).

## Results

Table 3 shows the predicted post-mortem ^239^Pu activities in the liver, skeleton, and liver+skeleton calculated using the three different sets of urine bioassay measurements–all urine measurements *A*_U_, urine collected during exposure *A*_U(E)_, and urine collected post-exposure *A*_U(P)_. All three sets included real measurements as well as measurements <MDA/2.

**Table 3:** Plutonium activities in the liver, skeleton, and liver+skeleton at the time of death predicted using urine bioassay.

Case	*A*_U_^a^ (Bq)			*A*_U(E)_^b^ (Bq)			*A*_U(P)_^c^ (Bq)		
	Liver	Skeleton	Liver+Skeleton	Liver	Skeleton	Liver+Skeleton	Liver	Skeleton	Liver+Skeleton
0060	110.8	149.0	259.7	121.3	163.1	284.4	108.2	145.5	253.8
0193	43.8	60.9	104.8	201.2	279.8	481.0	43.6	60.6	104.2
0255	17.7	25.2	42.9	167.4	238.3	405.7	17.1	24.3	41.4
0631	47.5	72.3	119.7	216.4	329.3	545.7	42.1	64.0	106.1
0634	49.8	76.8	126.7	192.7	296.9	489.5	45.4	69.9	115.3
0635	503.9	744.9	1248.8	1261.3	1864.4	3125.7	426.0	629.7	1055.8
0680	617.0	909.4	1526.4	743.0	1095.1	1838.1	605.2	892.0	1497.1
0719	145.5	213.7	359.2	391.7	575.5	967.2	131.2	192.7	323.9
0745	113.6	170.1	283.7	223.8	334.9	558.7	87.7	131.3	219.0
0769	116.8	166.9	283.7	217.8	311.4	529.2	107.5	153.7	261.2
0778	803.1	1146.7	1949.8	1711.3	2443.3	4154.6	675.5	964.5	1640.0

^a^Calculated using all urine data.

^b^Calculated using the subset of urine data collected during exposure.

^c^Calculated using the subset of urine data collected post-exposure.

**Fig 3:**
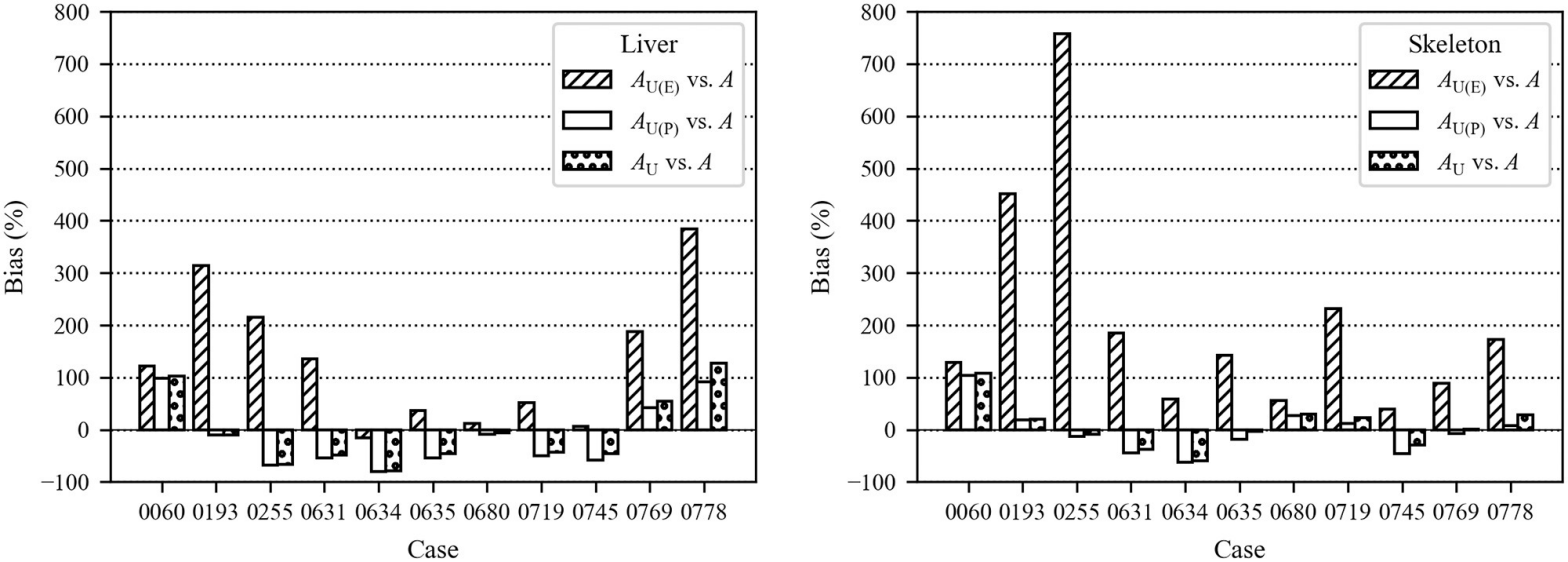
Bias in liver (left) and skeleton (right) activities predicted from urine bioassays.

**Fig 4:**
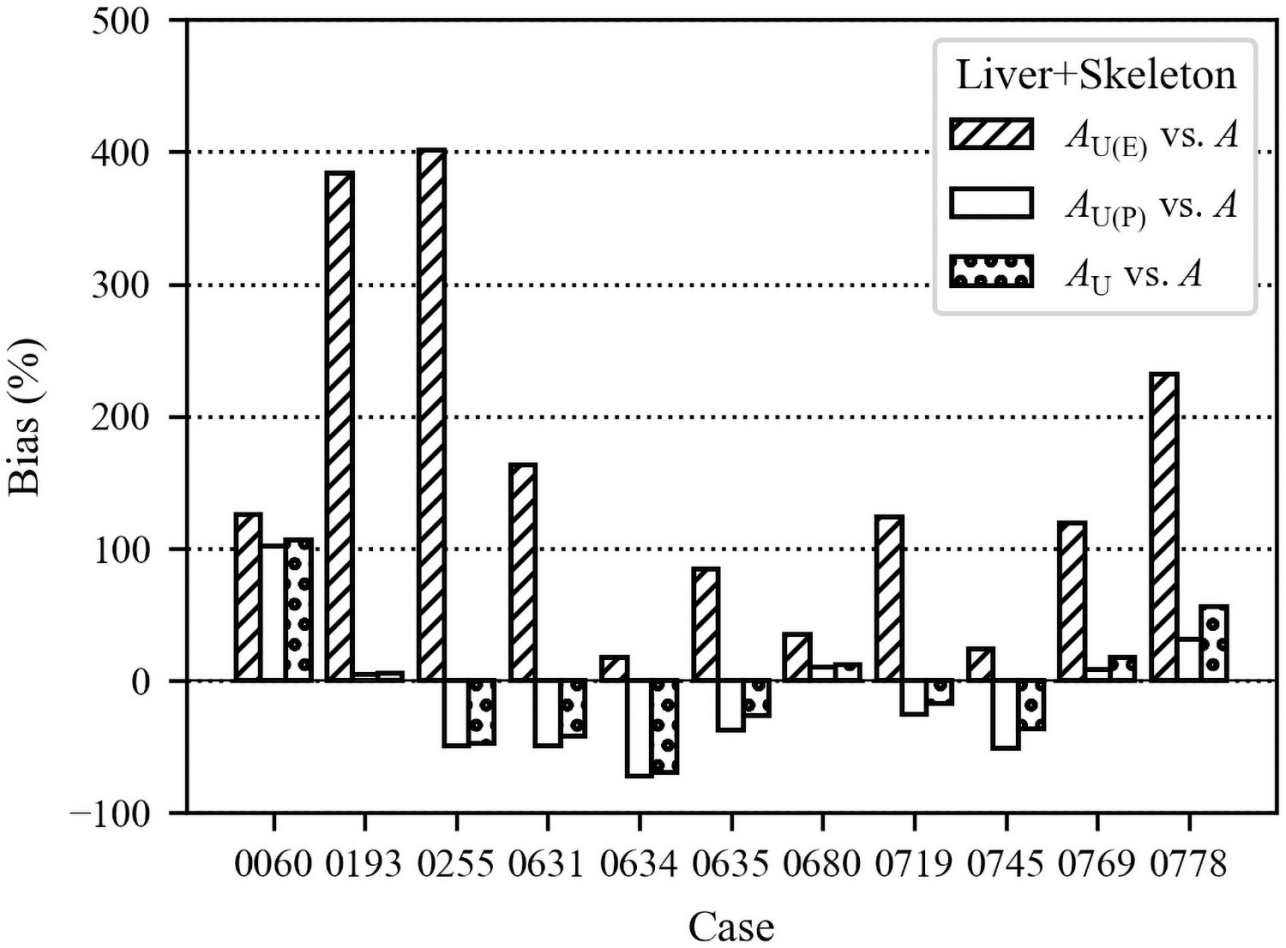
Bias in liver+skeleton activities predicted from urine bioassays.

Fig 3 shows the bias for the predicted and measured activities in the liver and skeleton with respect to measured activities. Fig 4 shows the bias for the sum of liver and skeleton activities (Eq 2).

Table 4 summarizes durations of chronic intakes and estimated *E*(50) that were predicted using all urine measurements *E*_U_, urine data collected during exposure *E*_U(E)_, post-exposure *E*_U(P)_, post-mortem activities in liver+skeleton *E*_LS_, and urine measurements and post-mortem activities simultaneously *E*_U+LS_. The longest chronic intake lasted for 1678 d for Case 0745, the shortest lasted 259 d for Case 0719. The average duration of chronic intake was 558 d, the median duration was 409 d. Moreover, Table 4 shows biases for the *E*(50) with *E*_U+LS_ as a reference (Eq 3).

**Table 4:** Committed effective doses calculated using five different sets of data.

Case	Exposure duration (d)	Committed effective dose *E*(50) (Sv)					Bias (%)	
		*E*_U_ ^a^	*E*_U(E)_ ^b^	*E*_U(P)_ ^c^	*E*_LS_ ^d^	*E*_U+LS_ ^e^	$\frac{E_{\text{U}}-E_{\text{U+LS}}}{E_{\text{U+LS}}}$	$\frac{E_{\text{U(E)}}-E_{\text{U+LS}}}{E_{\text{U+LS}}}$
0060	408	0.194	0.212	0.189	0.094	0.103	87.7	106
0193	452	0.083	0.382	0.083	0.079	0.083	0.8	363
0255	320	0.036	0.337	0.034	0.067	0.042	-14.7	707
0631	391	0.118	0.537	0.104	0.204	0.161	-26.7	234
0634	340	0.131	0.505	0.119	0.431	0.219	-40.4	131
0635	412	1.129	2.825	0.954	1.535	1.399	-19.3	102
0680	578	1.370	1.650	1.344	1.222	1.269	8.0	30.0
0719	259	0.320	0.861	0.288	0.385	0.366	-12.6	135
0745	1,678	0.265	0.522	0.205	0.421	0.363	-27.0	43.8
0769	409	0.238	0.444	0.219	0.202	0.211	12.5	110
0778	894	1.631	3.475	1.372	1.046	1.180	38.3	195

^a^Calculated using all urine data.

^b^Calculated using the subset of urine data collected during exposure.

^c^Calculated using the subset of urine data collected post-exposure.

^d^Calculated post-mortem activities in liver+skeleton.

^e^Calculated using all available data.

**Fig 5:**
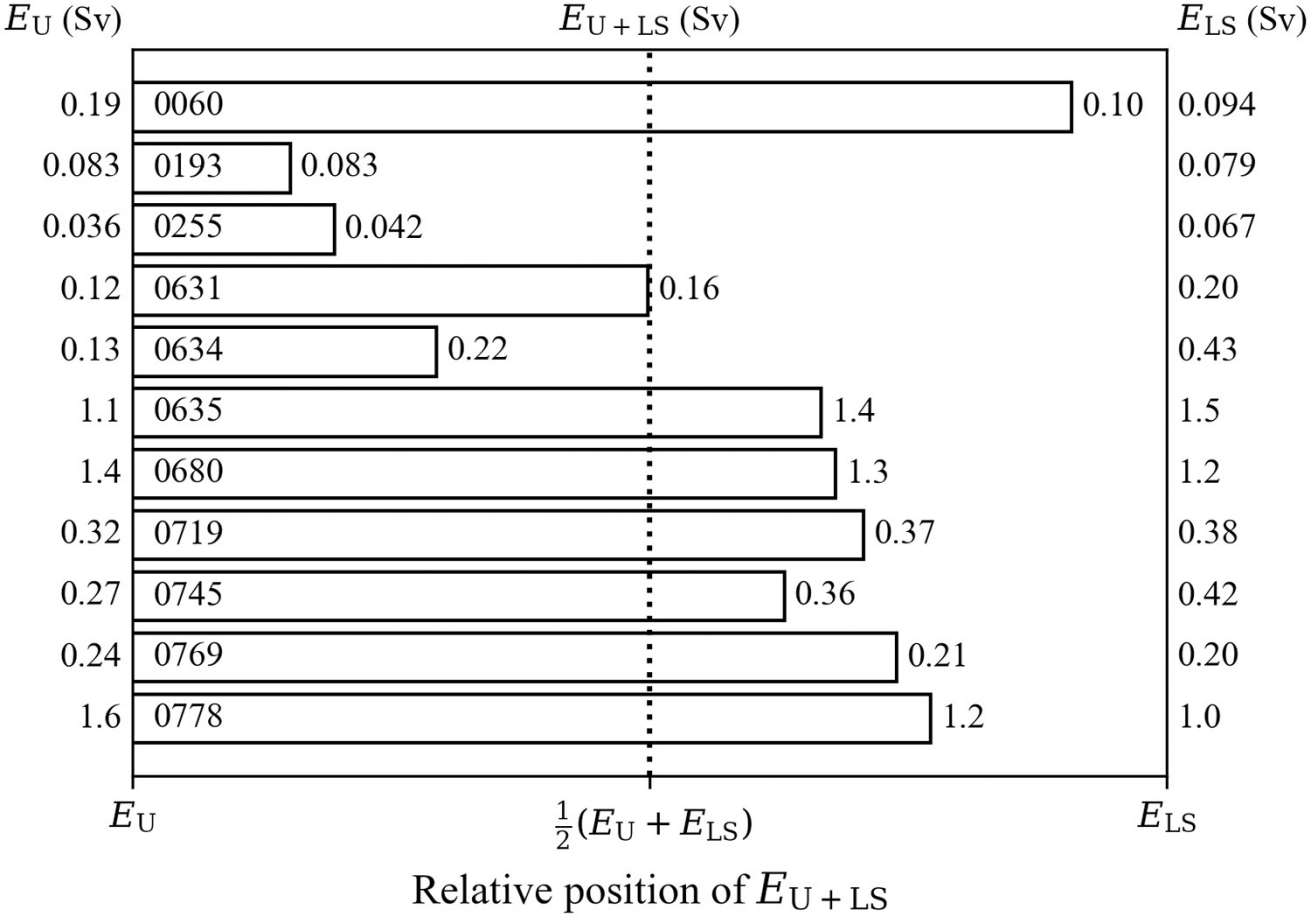
Position of committed effective dose *E*_U+LS_ between *E*_LS_ and *E*_U_ (Bq d^–1^). Vertical dotted line at 50% indicates the average of *E*_LS_ and *E*_U_.

Fig 5 shows which data, urine bioassay or the liver+skeleton activity, had more impact on dose predictions when fitting all data simultaneously. On the left side, there are doses predicted using urine data only (U), on the right side, there are doses predicted using the liver+skeleton activity only (LS). The bars represent a relative position of the prediction using all available data (U+LS) between urine-only and liver+skeleton-only predictions. If the bar is closer to the left side, the urine data had more impact on the fit. The closer the bar is to the right side, the more impact post-mortem activity measurements had.

Table 5 shows cumulative lifetime doses for time periods from the beginning of intake to death, and 50-y projected doses to the liver, red bone marrow, endosteal bone surface, and brain. The doses were calculated using intake predictions based on all available data, i.e., all urine bioassay results and post-mortem organ activities.

**Table 5:** Absorbed lifetime and 50-y projected doses to selected organs.

Case	Commitment period (y)	Lifetime dose (mGy)				50-y projected dose (mGy)			
		Liver	R-marrow^a^	Endost-BS^b^	Brain	Liver	R-marrow	Endost-BS	Brain
0060	29.8	23.1	2.3	19.6	0.18	35.4	3.0	27.8	0.29
0193	38.0	22.6	2.1	18.5	0.18	28.4	2.4	22.3	0.23
0255	43.7	12.8	1.1	10.3	0.10	14.3	1.2	11.3	0.12
0631	66.2	67.5	5.5	51.8	0.56	55.2	4.7	43.4	0.45
0634	72.5	98.1	7.8	74.8	0.81	75.3	6.4	59.3	0.61
0635	56.0	512.1	43.0	399.6	4.18	480.5	41.1	377.4	3.90
0680	54.2	458.6	38.6	358.5	3.74	435.9	37.2	342.4	3.54
0719	52.7	130.1	11.0	101.9	1.06	125.6	10.7	98.6	1.02
0745	61.2	141.0	11.7	109.2	1.15	124.8	10.7	98.0	1.01
0769	44.9	66.2	5.8	52.9	0.53	72.6	6.2	57.0	0.59
0778	45.3	367.8	32.4	293.8	2.97	405.1	34.6	318.2	3.29

^a^Red marrow.

^b^Endosteal cells (bone surface).

## Discussion

### Post-mortem organ activities

A comparison of activities predicted using urine bioassay data serves as a test of the quality of the biokinetic model. In workplace radiation exposure monitoring, urine bioassays are routinely analyzed during a potential exposure period. This situation was represented by activity *A*_U(E)_, for which the mean absolute biases (± sample standard deviation) for the liver and skeleton activities were 135±129%, and 210±215%, respectively. Predictions based on post-exposure urine bioassays, *A*_(P)_, were used to show the influence of follow-up measurements on the results. When post-exposure measurements were used to predict organ activities at the time of death, *A*_U(P)_, the mean absolute biases were 56±29% and 33±30% for the liver and skeleton, respectively. When all available urine date, *A*_U_, were used, the mean absolute biases were 57±36% and 32±30% for the liver and skeleton, respectively. Thus, the post-exposure urine bioassay dominated the estimates.

**Fig 6:**
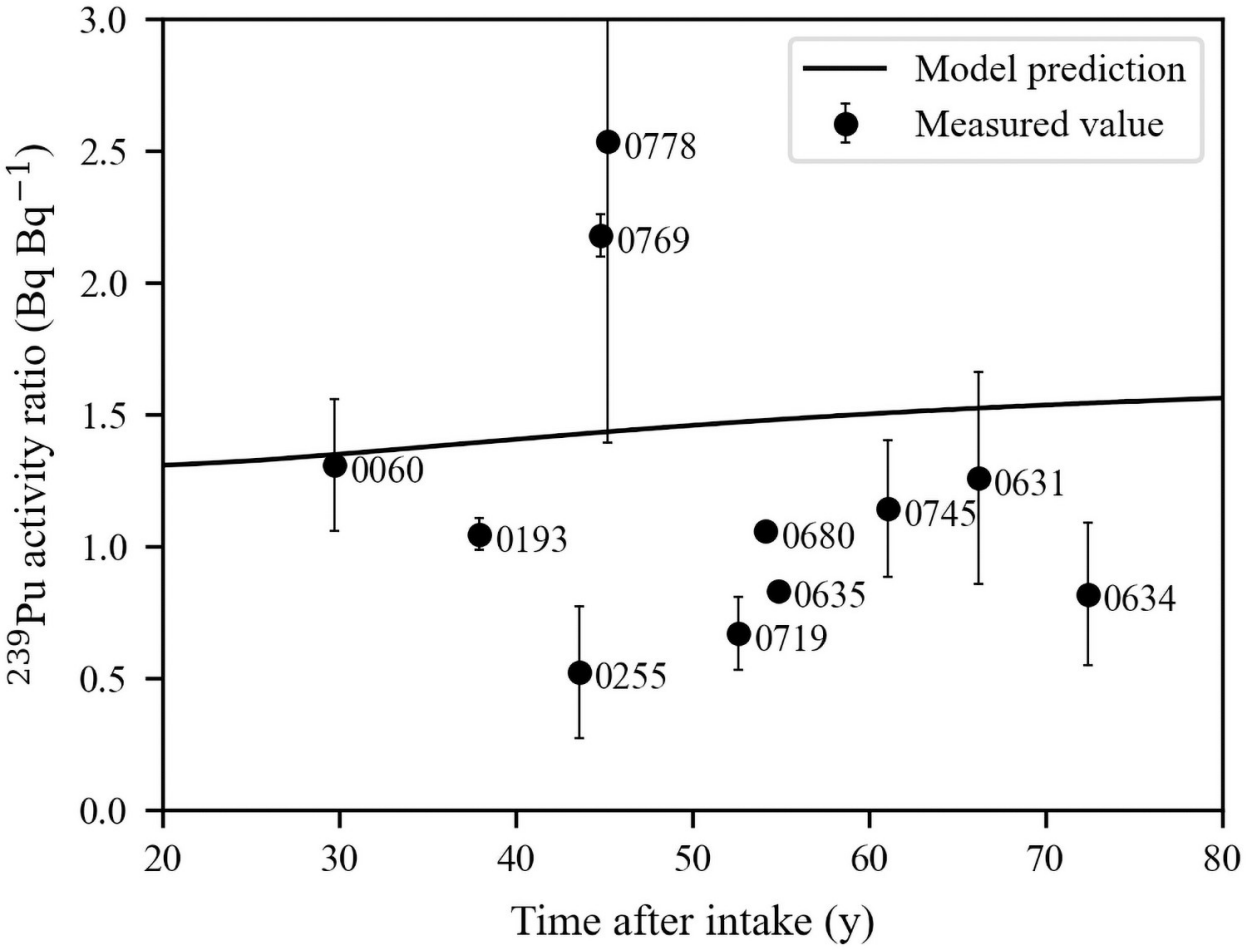
Skeleton-to-liver ^239^Pu activity ratio.

As follows from Fig 3, the biases in the liver and skeleton activities differed significantly from each other due to individual variability of systemic activity distribution between the liver and skeleton. This is often represented as a skeleton-to-liver activity ratio. The biokinetic model predictions of this ratio in years post-intake and the skeleton-to-liver ratio at the time of death for this group are shown in Fig 6. The figure shows that nine cases fall below the curve, meaning that the actual skeleton activity in comparison to the liver activity was lower than the model predicted. The geometric mean of the ratio of skeleton to liver post-mortem activity was 1.10 with a GSD of 1.60. According to the ICRP systemic model for plutonium [38], the skeleton-to-liver activity ratio 10,000 *d* after intake equals 1.33, and 20,000 *d* after intake equals 1.48 [38]. In the Mayak worker study, the average skeleton-to-liver activity ratios of 1.2, 1.9, and 5.5 were reported for healthy individuals, chronically ill individuals with no liver pathology, and individuals with liver diseases such as cirrhosis or carcinomas, respectively [56,57].

Biases for the liver+skeleton activity are shown in Fig 4. The mean absolute biases were 156±133%, 40±29%, 40±30% for *A*_U(E)_, *A*_U(P)_, and *A*_U_, respectively. The biases for *A*_U(E)_ were the largest and were all positive. This means that use of urine data collected for these individuals during exposure overestimated the total systemic content on average by 156%. The poor quality of early urine monitoring data likely contributed to the overestimation. Riddell et al. [8] expressed a lack of confidence in early urine data. Due to a high potential of sample contamination, as well as significant changes in the monitoring program, the authors of that paper had excluded data obtained before 1971, wherever possible. Our data showed a similar trend since later follow-up urine bioassays provided substantial improvement in the accuracy of systemic activity estimates. Early urine measurements were not reliable for various reasons, including the high background of detectors, low sensitivity, gross alpha counting instead of alpha spectrometry, and no individual yield corrections to account for losses during the radiochemical process [58,59]. Moreover, only several urine measurements were obtained during the relatively short exposure periods for these cases (Figs 2 and 3). Therefore, the knowledge of the precise period of the chronic intake or the acute intake time(s) is crucial for a reliable intake estimate from early measurements since urine excretion varies the most during the first weeks after the intake. For urine collected years after the exposure, the precision of the intake time or period does not impact the estimated intake activity.

The mean liver+skeleton-activity biases were –12±50%, and –4±51% for *A*_U(P)_, and *A*_U_, respectively. Therefore, on average, for these 11 individuals, the model prediction of ^239^Pu systemic activity using post-exposure or all urine data was in good agreement with the measurements. Predictions of post-mortem activities in the liver+skeleton were improved by including post-exposure urine measurements. Post-exposure urine measurements had a higher impact because there were more of them, and their GSDs were lower.

The importance of urine follow-up measurements was especially demonstrated for Case 0193. This case had the second-largest bias (almost 400%) for *A*_U(E)_, but the lowest for *A*_U(P)_ and *A*_U_ (4.8% and 5.4%). The largest difference for all urine data was recorded for Case 0060, who died 30 y after exposure (Table 5), and for whom only three urine post-exposure measurements were available (Fig 2). For Case 0060, the activity predicted from urine was approximately twice as high as the measured activity.

### Radiation doses

#### Committed effective doses

The committed effective dose estimates based on early urine bioassays, *E*_U(E)_, differed significantly from the doses that were estimated using all available information, *E*_U+LS_ (Table 4)–on average by 196±193%. For all cases, the biases were positive, i.e., *E*_U(E)_>*E*_U+LS_. The largest bias was observed for Case 0255 (707%); the lowest for Case 0680 (30%). This further demonstrated the utility of post-exposure follow-up measurements.

The mean absolute bias for *E*_U_ was 26.2±23.8%, the mean bias value was 0.6%. For two cases (Cases 0193, 0680), *E*_U_ were within 10% of *E*_U+LS_, for four, between 10 and 20% (Cases 0255, 0635, 0719, 0769). For Case 0193, the agreement of *E*_U_ and *E*_U+LS_ was exceptional (0.8%). The largest bias between *E*_U_ and *E*_U+LS_ was observed for Case 0060 (87.7%) and 0634 (–40.4%). For Case 0634, two urine data points in Fig 2 below 0.001 Bq d^–1^ appeared to be outliers. If these two urine measurements were excluded, the committed effective doses would be *E*_U_ = 0.196 Sv and *E*_U+LS_ = 0.320 Sv. This increased the dose estimate, and decreased the bias of *E*_U_ vs. *E*_U+LS_ (–38.6%). No skeleton, liver, or kidney pathology was found in medical records of Case 0634 to explain the difference. If this individual were excluded from the studied group, the mean absolute bias for *E*_U_ vs. *E*_U+LS_ would slightly decrease to 24.8±24.6%.

Committed effective doses predicted using both urine bioassay and post-mortem activity, *E*_U+LS_, fell between doses predicted using only the liver+skeleton post-mortem activities, *E*_LS_, and doses predicted using only urine bioassay, *E*_U_. Fig 5 shows the relative position of *E*_U+LS_ between *E*_U_ and *E*_LS_. The longer the bar is, the closer *E*_U+LS_ is to *E*_LS_. For Case 0631, *E*_U+LS_ bar ends at the midpoint, meaning the urine measurements and the liver+skeleton activity had equal weight in the fit. Cases 0193 and 0255 had the largest number of urine measurements, therefore, *E*_U+LS_ is closer to *E*_U_, even though the difference between *E*_U_ and *E*_LS_ was very narrow for Case 0193. For Case 0634, the position of *E*_U+LS_ was highly influenced by the two very low urine measurements discussed above; excluding them moved the bar closer to *E*_LS_. The actual positions of *E*_U+LS_ between *E*_U_ and *E*_LS_ tended to be affected by the number of urine measurements (the more urine measurements, the closer *E*_U+LS_ was to *E*_U_), the error models and values of standard deviation or the GSD used for the fit of data, as well as the difference between *E*_U_ and *E*_LS_. Therefore, it is crucial to choose appropriate error values when predicting intake from multiple bioassay data and post-mortem measurements.

#### Lifetime organ doses

On average, the actual commitment period of 51.3±12.6 y is essentially the same as the standard 50 y assumption used for the committed equivalent and effective dose calculations. However, the commitment periods for individuals differed from 50 y leading to different lifetime doses. Six cases lived longer than 50 y after exposure; for example, Case 0634 lived the longest, 72.5 y after exposure, increasing the dose to the liver, red bone marrow, bone surface, and brain by 30%, 22%, 26%, and 32%, respectively. Conversely, five cases lived less than 50 y after exposure. For Case 0060, who lived the shortest (29.8 y) after the intake, the actual doses to the liver, red marrow, bone surface and brain were 35%, 25%, 30%, and 27% lower than the 50-y dose. Therefore, as also follows from Table 5, the liver and brain appear to be slightly more sensitive to the length of the commitment period than bones. For all studied individuals, the mean lifetime doses to the liver, red marrow, bone surface, and brain were 173, 14.7, 136, and 1.41 mGy, while the mean 50-y doses were 168, 14.4, 132, and 1.37 mGy, respectively.

## Conclusion

Biases for the predicted and measured post-mortem organ activities and calculated radiation doses, caused by differing types and times of urine measurements were investigated as a pilot study of 11 former Manhattan Project workers. This group provided a unique dataset, due to both the availability of post-mortem organ measurements made by the USTUR, and post-exposure urinalysis data collected by their worksite medical follow-up program. Post-mortem activities in the liver and skeleton, effective doses, and absorbed doses to selected organs were calculated using urine bioassay data. On average, current biokinetic model predictions for the liver+skeleton retention appear to be in good agreement with the measured organ activities (–4±51%); however, the individual variability is high. Use of urine bioassay data collected during the exposure period in the 1940s overestimated the liver+skeleton activity on average by a factor of 2.5, likely due to poor quality of early urine measurements. Therefore, caution is advised when radiation epidemiological studies calculate organ doses from urine bioassays collected in the 1940s. Using post-exposure urinalyses significantly improved the estimates of organ activities and doses. This demonstrates the importance of a long-term collection of bioassays as a part of follow-up.

## Supporting information

S1 Appendix Urine bioassay data. (XLSX)

S2 Appendix Bone samples for partial body cases. (XLSX)
